# Supplementary figures and images for: ANK2 Hypermethylation in Canine Mammary Tumors and Human Breast Cancer
Source: Int J Mol Sci. 2020 Nov 18;21(22):8697. doi: 10.3390/ijms21228697 (PMC7698701; doi:10.3390/ijms21228697)

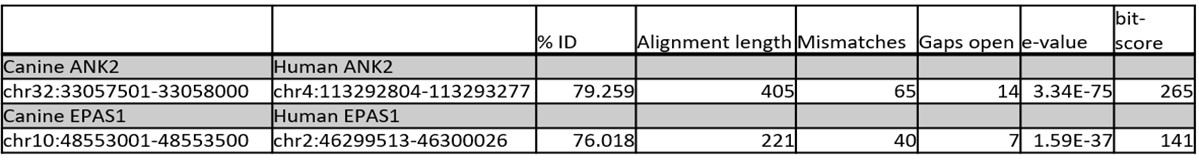

Supplement: Supplementary file 1 [file ijms-21-08697-s001.zip › Table S1. Sequence Homology of target Canine ANK2 and EPAS1 regions with their orthologous human regions..jpg]

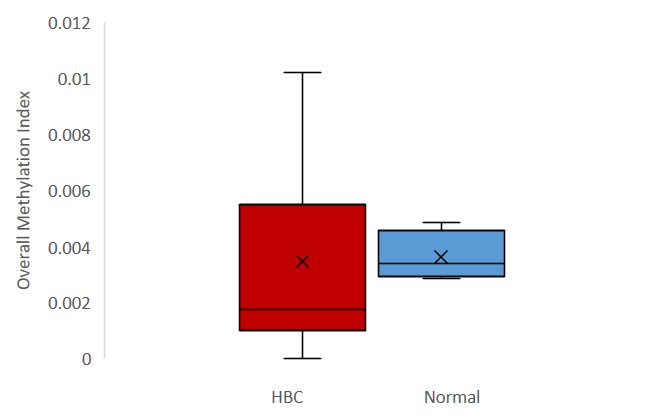

Supplement: Supplementary file 1 [file ijms-21-08697-s001.zip › Figure S1. qMSP of ANK2 in HBC cfDNA.jpg]

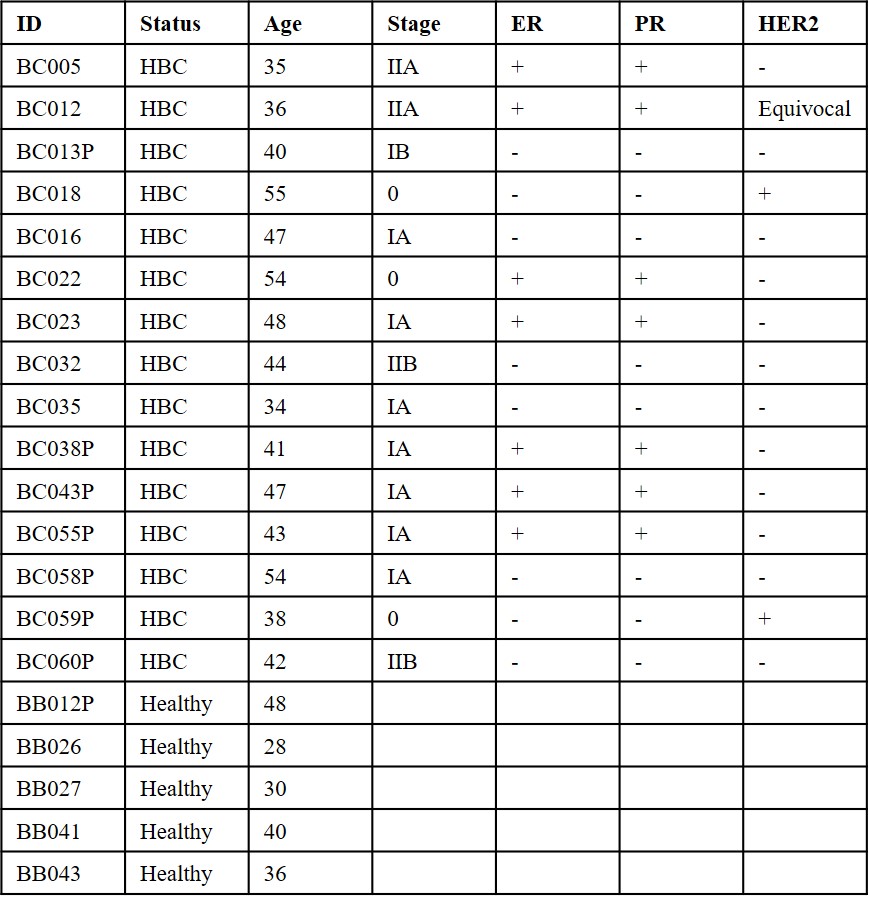

Supplement: Supplementary file 1 [file ijms-21-08697-s001.zip › Table S3. Female Breast Cancer and normal plasma samples..jpg]

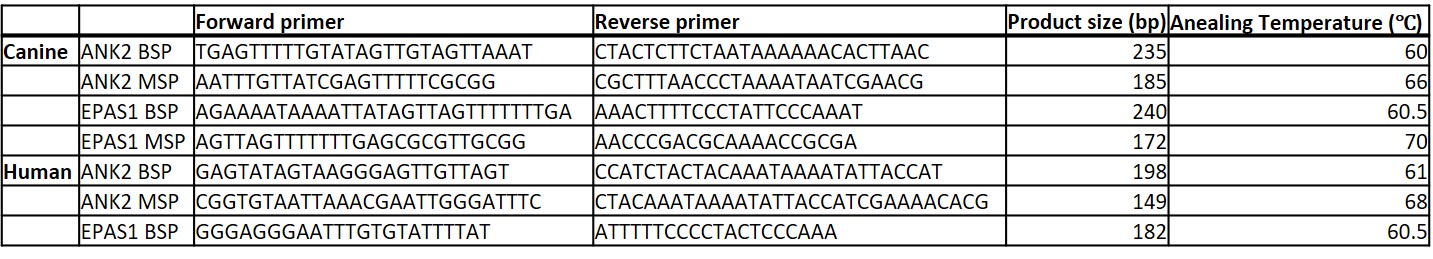

Supplement: Supplementary file 1 [file ijms-21-08697-s001.zip › Table S4. Primer sets for BSP and MSP.jpg]

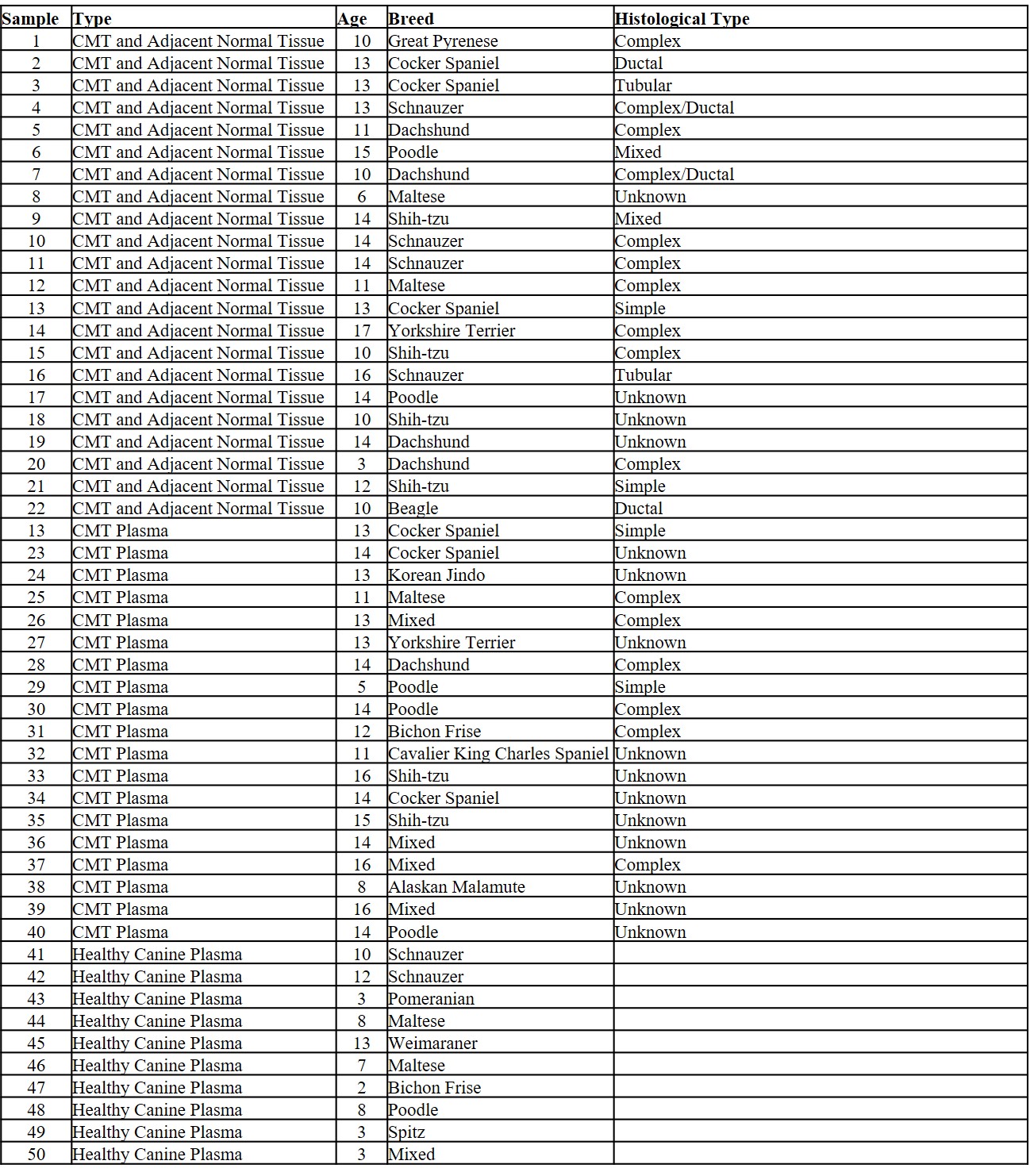

Supplement: Supplementary file 1 [file ijms-21-08697-s001.zip › Table S2. Canine tissue and plasma data.jpg]
